# Mortality from Alcoholic Cardiomyopathy: Exploring the Gap between Estimated and Civil Registry Data

**DOI:** 10.3390/jcm8081137

**Published:** 2019-07-31

**Authors:** Jakob Manthey, Jürgen Rehm

**Affiliations:** 1 Institute of Clinical Psychology and Psychotherapy, TU Dresden, Chemnitzer Str. 46, 01187 Dresden, Germany; 2 Center for Interdisciplinary Addiction Research (ZIS), Department of Psychiatry and Psychotherapy, University Medical Center Hamburg-Eppendorf (UKE), Martinistraße 52, 20246 Hamburg, Germany; 3 Institute for Mental Health Policy Research, CAMH, 33 Russell Street, Toronto, ON M5S 2S1, Canada; 4 Dalla Lana School of Public Health, University of Toronto, 155 College Street, 6th Floor, Toronto, ON M5T 3M7, Canada; 5 Campbell Family Mental Health Research Institute, CAMH, 250 College Street, Toronto, ON M5T 1R8, Canada; 6 Institute of Medical Science (IMS), University of Toronto, Medical Sciences Building, 1 King’s College Circle, Room 2374, Toronto, ON M5S 1A8, Canada; 7 Department of Psychiatry, University of Toronto, 250 College Street, 8th Floor, Toronto, ON M5T 1R8, Canada; 8 WHO Collaborating Centre for Mental Health and Addiction, 33 Russell Street, Toronto, ON M5S 2S1, Canada; 9 Department of International Health Projects, Institute for Leadership and Health Management, I.M. Sechenov First Moscow State Medical University, Trubetskaya str., 8, b. 2, Moscow 119992, Russia

**Keywords:** alcoholic cardiomyopathy, cardiovascular diseases, GBD study, vital statistics, garbage code, cause of death, alcohol per capita consumption, mortality, heart failure

## Abstract

Background: Based on civil registries, 26,000 people died from alcoholic cardiomyopathy (ACM) in 2015 globally. In the Global Burden of Disease (GBD) 2017 study, garbage coded deaths were redistributed to ACM, resulting in substantially higher ACM mortality estimates (96,669 deaths, 95% confidence interval: 82,812–97,507). We aimed to explore the gap between civil registry and GBD mortality data, accounting for alcohol exposure as a cause of ACM. Methods: ACM mortality rates were obtained from civil registries and GBD for *n* = 77 countries. The relationship between registered and estimated mortality rates was assessed by sex and age groups, using Pearson correlation coefficients, in addition to comparing mortality rates with population alcohol exposure—the underlying cause of ACM. Results: Among people aged 65 years or older, civil registry mortality rates of ACM decreased markedly whereas GBD mortality rates increased. The widening gap of registered and estimated mortality rates in the elderly is reflected in a decrease of correlations. The age distribution of alcohol exposure is more consistent with the distribution of civil registry rather than GBD mortality rates. Conclusions: Among older adults, GBD mortality estimates of ACM seem implausible and are inconsistent with alcohol exposure. The garbage code redistribution algorithm should include alcohol exposure for ACM and other alcohol-attributable diseases.

## 1. Introduction

Heavy alcohol use is a major contributor to cardiovascular diseases (CVD) [1,2]. Among other conditions, high levels of alcohol use can lead to alcoholic cardiomyopathy (ACM), which is characterized by a dilation and impairment of the left ventricle [3]. While this condition is the result of the toxic effects of alcohol, the symptoms and clinical presentation are not unique to ACM but largely resemble other dilated cardiomyopathies [4]. As with other cardiomyopathies, ACM is associated with systolic dysfunction and a considerable risk factor for myocardial infarctions and sudden death [4,5]. In the past few years, interest on ACM grew as reflected in the publication of several reviews describing clinical presentations [5], pathophysiological mechanisms [6], as well as the causal relationship between heavy alcohol consumption and incidence of ACM [7]. 

For most alcohol-attributable conditions, mortality is estimated via alcohol-attributable fractions (AAFs) based on alcohol exposure and risk relations. For ACM, however, this method cannot be applied to due to a lack of data quantifying the risk relations [7]. Hence, an alternative method was proposed [8], based on cause of death data reported by countries with civil registration of vital statistics [9]; these vital statistics were available for 91 countries and formed the basis to model ACM as cause of death for all countries and globally [10]. According to these estimates, there were 25,997 global deaths from ACM in 2015. Applying a different methodology, the Global Burden of Disease (GBD) 2017 study resulted in a more than three-fold mortality figure (90,669 deaths in 2015, 95% confidence interval: 82,812–97,507) [11]. Higher GBD estimates were the result of redistributing so called garbage coded deaths to well-defined cause of death codes, including ACM and other CVD [12]. The term garbage code has first been coined in 1996 and refers to all codes, which are not useful for public health analyses [13,14]. More precisely, the term encompasses all codes that are not recognized by the Tenth revision of the International Classification of Diseases (ICD-10, [15]) in describing the actual underlying cause of death. Instead, garbage codes may indicate a symptom (e.g., pain) or an intermediate cause of death, such as heart failure [16].

The outlined gap in ACM mortality estimates warrants further attention given the large difference and because GBD mortality estimates are susceptible to the methods employed in redistributing garbage coded deaths, as previously demonstrated for ischemic heart disease [17]. In this contribution, we sought to further explore the gap between registered and estimated ACM deaths. For this purpose, we used mortality data from civil registries, which serve as one main input for the GBD study, and compare these data with mortality estimates as published in the last GBD update [12]. We aimed to examine the association of registered and estimated ACM mortality rates in countries with available civil registry data. Further, we compared ACM mortality distributions to the distribution of alcohol exposure—its underlying cause by definition. In sensitivity analyses, we examined associations of ACM and garbage coded deaths, which constitute the base for redistributing garbage codes to well-defined causes of death in the GBD study [12]. 

## 2. Experimental Section

### 2.1. Description of Data Sources and Disease Definitions

We obtained adult (15 years or older) mortality data from two sources: (1) civil registration data from the World Health Organization (WHO) mortality database [18], and (2) estimated mortality data from the GBD 2017 study [11]. 

From the WHO mortality database, we retained all country-years with any four-digit ICD-10 code and available data on sex and age. After matching the ICD-10 codes [15] with the disease categories as defined in the GBD 2017 study, we obtained country-, year-, sex-, and age-specific mortality data for the following categories: CVD, cardiomyopathy and myocarditis (hereafter referred to as ‘all cardiomyopathies’), ACM, as well as CVD and heart failure garbage codes (for the definition of each category, see Appendix A and [19]). We calculated the death counts for a new cause of death category ‘all CVD’ from the sum of CVD and CVD garbage code cause of death definition. For country-years with mortality data available only for one sex, we assumed 0 deaths for the other sex. From the GBD database, we obtained the estimated mortality data for the same disease categories (except for garbage codes) [11].

The two mortality data sets were then matched on the above given disease categories and all country-years with available mortality data on ACM and heart failure garbage codes from civil registries were retained, resulting in a total number of 77 countries (with 823 country-years). In order to calculate mortality rates (i.e., deaths per 100,000 adults) we combined the mortality data with population estimates from the UN Population Division [20]. Alcohol exposure was defined as intake of pure alcohol per adult (in liters per year, alcohol *per capita* consumption). Alcohol exposure data were obtained from a recent modeling study using WHO sources and forecasting techniques [21]. Alcohol exposure data was not available for all 5-year age bands, which required the aggregation of mortality data into the available age groups. In addition to same-year, 5-year and 10-year lagged alcohol exposure estimates were calculated to account for the estimated lag time between exposure and disease incidence [7]. These lag times are in line with findings of a recent review and clinical guidelines [7,22].

### 2.2. Descriptive Analyses

For descriptive analyses, we only used data from the most recent available year per country (*n* = 77 data points by sex and age group). For both registered and estimated deaths, we calculated death counts and age-standardized mortality rates per 100,000 adults. To illustrate the gap between registered and estimated deaths, we calculated the ratio of estimated to registered deaths and mortality rates (the larger the ratio, the larger the gap). To examine the association of registered and estimated ACM deaths, Pearson correlations were computed for all adults and by sex and age. 

### 2.3. Sensitivity Analyses

In sensitivity analyses, we aimed to test whether ACM deaths and garbage coded deaths were negatively associated. In the GBD study, a negative association between garbage coded deaths and target diseases is the requirement for the redistribution models [12,13,23]. In brief, these models assume that in jurisdictions with accurate coding practice, a low proportion of deaths are assigned to garbage codes and all other diseases are accurately coded. Consequently, a negative association indicates that the fewer deaths being assigned to garbage codes, the more deaths are being accurately coded. In the GBD study, a negative association between garbage coded deaths and a given cause of death is used as indicator for the redistribution of garbage coded deaths, while positive or non-significant associations indicate that garbage coded deaths may not be redistributed to the given disease.

In this contribution, we performed similar regression models using proportion of ACM deaths among all CVD deaths as target disease and proportion of heart failure deaths among all CVD deaths as garbage codes. We selected heart failure deaths as they were cited as source for redistributing deaths to ACM in the GBD 2017 study [12]. Furthermore, in previous studies the redistribution of heart failure deaths has resulted in an increase of deaths attributable to cardiomyopathy [16,23], which should theoretically increase the number of ACM deaths, as well. 

Poisson regression models examined the relationship of heart failure garbage coded deaths (independent variable) and ACM deaths (dependent variable), allowing for random intercepts in each country. As opposed to GBD redistribution models, we included alcohol *per capita* consumption as additional covariate, which was identified as main driver for estimating ACM mortality [10]. Further, we allowed for nonlinear associations by including polynomials of the independent variable. For more details on the sensitivity analyses, see Appendix A.

All analyses were conducted with *R* version 3.5.1 [24]. 

## 3. Results

A descriptive summary of the mortality data compiled for this study can be found in Table 1. 

### 3.1. Epidemiology of Registered and Estimated ACM Mortality

Using data from the most recent available year for each country, data from *n* = 77 countries could be obtained, representing 1.54 billion adults, mainly living in the Americas and WHO European Region (for a summary on included countries key data, see Supplementary Table S1). Since 1990, 63,016 ACM deaths (females: 8816; males: 54,200) were recorded in civil registries. For the same set of countries during the same period, the GBD study estimated the ACM death count at 370,675 (females: 66,080; males: 304,595). Thus, for each registered ACM death, nearly 5 additional deaths have been estimated in the GBD study for the included countries (female ratio: 7.5; male ratio: 5.6). For all cardiomyopathies, the gap between estimated to registered deaths is similar to ACM (female ratio: 8.1; male ratio: 6.3), but it is considerably lower for deaths from all CVD (female ratio: 1.8; male ratio: 1.6).

**Figure 1:**
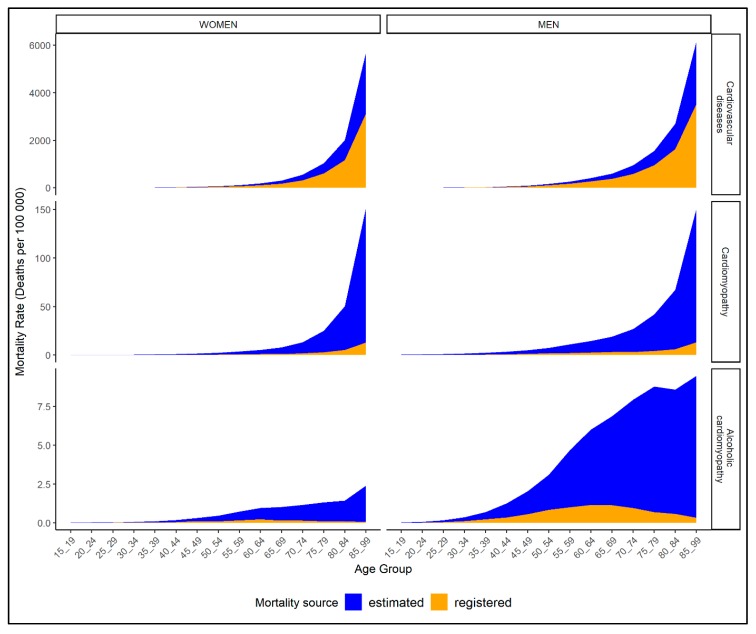
Mortality rates of registered (orange) and estimated (blue) deaths over the life span by cause of death definition (rows) and sex (columns); based on most recent available mortality data from *n* = 77 countries.

While the variation by sex was relatively low, there were substantial differences in the distribution of estimated and registered deaths across all ages by cause of death definition. In Figure 1, the registered and estimated mortality rates are presented by sex and across all 5-year age groups (see Appendix A for mortality rates by cause, sex, and age). The age distribution of registered and estimated deaths was largely parallel for CVD, with largely constant ratios of estimated to registered mortality rates across all age groups (1.5–2.0). For all cardiomyopathies, distribution of registered and estimated mortality rates were similar, with exponential increases in older age groups. The ratios of estimated and registered mortality rates for all cardiomyopathies were closer among 15 to 59 year olds (2.4–4.7) and increased in older ages (75 years or older: 9.1–11.4). 

In contrast, the age distribution of registered and estimated mortality rates of ACM diverged substantially. While registered mortality rates largely resemble a normal distribution peaking at ages 60–64 (0.7 deaths per 100,000 adults) and decreasing thereafter, the estimated ACM mortality rates were left-skewed and peaked in the oldest age group (4.7 deaths per 100,000 people aged 85+ years). Consequently, the gap between estimated and registered mortality rates widened with increasing age, with lowest ratios among 25 to 64 year olds (3.2–5.0) and highest ratios in older ages (75 years and older: 13.1–33.4).

**Figure 2:**
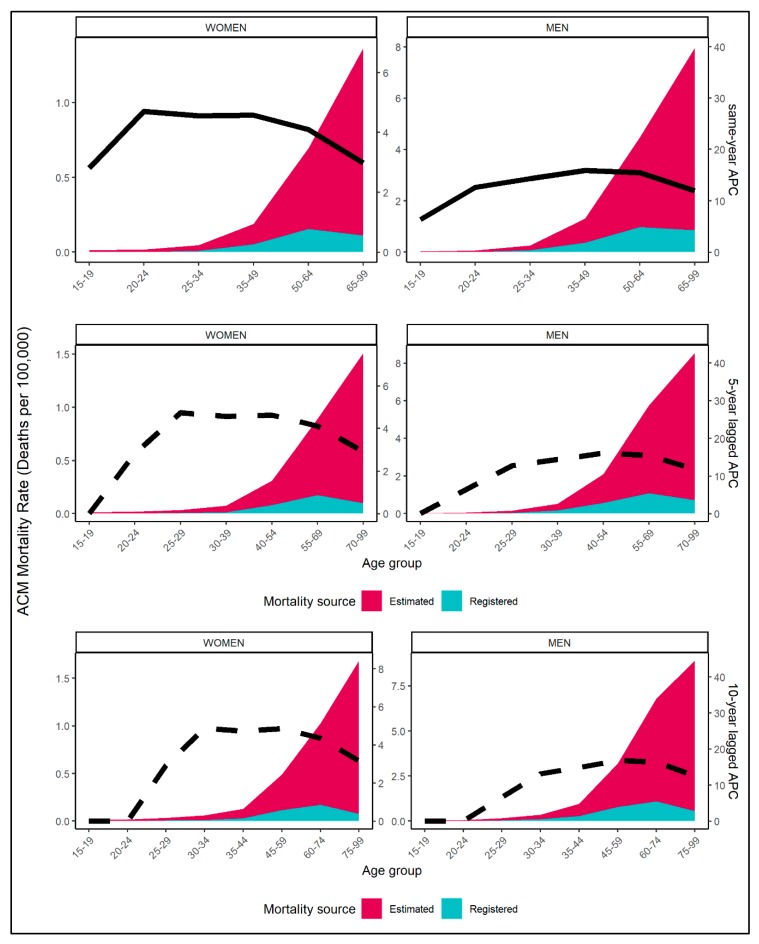
Estimated (red) and registered (blue) ACM mortality rates per 100,000 and alcohol per capita consumption (APC) over selected age groups and by sex (column) for most recent available data of *n* = 77 countries; solid line denotes same-year APC (first row) and dashed line denotes 5-year (second row) and 10-year lagged APC (third row).

The diverging age pattern in ACM mortality figures can also be observed in Figure 2, where registered and estimated mortality rates are presented together with alcohol exposure estimates, for available age groups. The plot suggests that the age distribution of alcohol exposure was more congruent with registered rather than estimated ACM mortality rates. Among older age groups, both registered mortality rates for ACM and alcohol exposure decreased, while the estimated mortality rates increased. Similar patterns can be observed for both same-year, 5-year, and 10-year lagged alcohol exposure.

The age-dependent association of registered and estimated ACM deaths is also presented in Table 2. The correlation of registered and estimated ACM mortality rates was high among all adults for both women and men. Among men, high correlations (>0.55) can be observed for all ages between 20 and 79 years. In older age groups, the correlations were below 0.20. Among women, registered and estimated ACM mortality rates were not associated in the youngest (25–29 years) and oldest (75 and older) age groups, but were associated in the age groups in between. Furthermore, and among both sexes, the association was most pronounced (i.e., >0.7) up to 64-year old people and decreases thereafter.

### 3.2. Sensitivity Analyses

**Figure 3:**
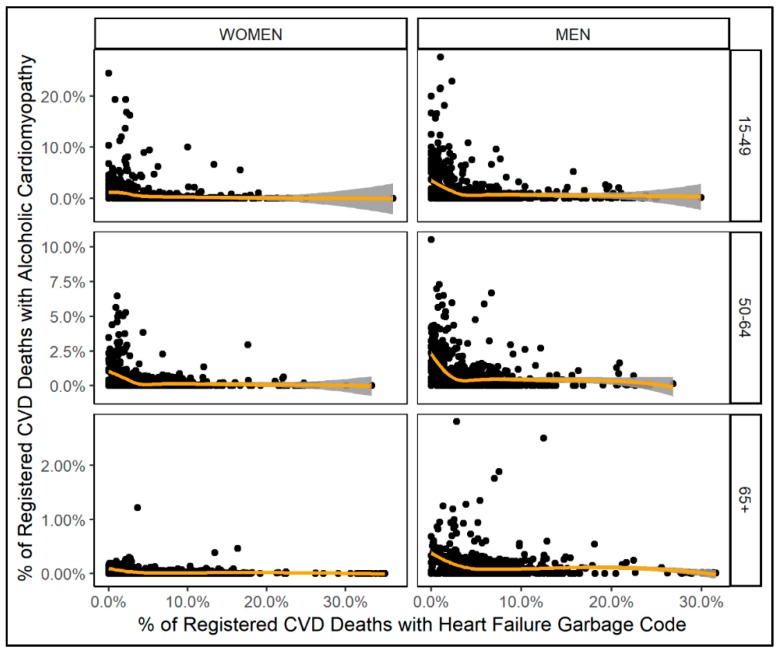
Scatter plots of proportion of heart failure garbage code deaths and proportion of ACM deaths among all CVD deaths by sex (column) and age group (row); orange line denotes the smoothing function of fitted proportion of ACM deaths among all CVD deaths obtained from multi-level models.

Results of sensitivity analyses are illustrated in Figure 3 (for model results, see Appendix A). Between proportion of ACM deaths and proportion of heart failure deaths among all CVD deaths, there was a non-monotonous negative association. Only for very low proportions of heart failure deaths among all CVD deaths (below 5%) could a decrease in the proportion of ACM deaths among all CVD deaths be observed.

## 4. Discussion

### 4.1. Summary of the Findings

This study compared mortality data from civil registries with estimates from the GBD 2017 study, using data from *n* = 77 countries.

The GBD mortality estimates of ACM seem implausible for the elderly population. Among people aged 65 years or older, the registered ACM mortality rates follow a decrease in alcohol exposure, which is the core determinant of ACM. A similar age distribution of ACM has also been identified in a recent study examining hospitalization data of the United States of America [25]. In contrast, the estimated ACM mortality rates continue to increase with an ageing population. Given a decrease in alcohol exposure among the elderly, an increase of ACM mortality in people aged 65 years or older is unlikely and may result in overestimating the total number of ACM deaths in the GBD study.

### 4.2. Improving ACM Mortality Estimates

For other alcohol-attributable diseases, mortality is estimated using alcohol-attributable fractions, which are calculated from exposure and risk functions (for global mortality estimates, see reference [26]; for a summary of risk functions, see reference [27]). As alcohol-attributable fractions are stratified by age groups, the decline in exposure in the elderly population can be reflected in lower alcohol-attributable fractions in these age groups. For ACM, mortality in the GBD study is estimated by redistributing garbage coded deaths, yet without accounting for age variations in alcohol exposure [12,23]. 

In our view, ACM mortality estimates should be made consistent with alcohol exposure, because this is its core determinant. Further, reductions of alcohol use have been associated with improvements of the clinical course of ACM [28], including mortality risks [29]. In order to align alcohol exposure and ACM mortality estimates in the GBD study, alcohol exposure data should be included in models estimating redistributing proportions for ACM. As indicated in this study, a 5-year or 10-year lag of alcohol exposure may prove useful in redistribution models, which likely represents the period of heavy alcohol intake required to develop ACM [7,22]. While a 5-year period of heavy chronic drinking has been used as lower bound to develop ACM [30,31], up to 25 years of heavy chronic drinking among affected patients have been reported in a number of clinical studies [30,32,33]. However, such long lag times may capture treatment onset rather than disease incidence, similar to the delay between onset and treatment of alcohol use disorders [34]. Thus, in the absence of data from population studies, we proposed to use a 10-year lag to model disease onset until further confirmative data is available. Among 15–24 year olds, a 10-year lag would result in 0 ACM deaths, which is largely in line with the registered deaths and is coherent with alcohol-attributable mortality estimation for cancer [26].

### 4.3. The Impact of Garbage Code Redistribution for ACM Mortality Estimates

In the GBD study, garbage coded deaths are redistributed to other well-defined diseases, including ACM. Since GBD 2013, redistribution proportions are estimated to redistribute garbage coded deaths to selected target diseases (the method proposed in reference [23]; for details of application see Appendix 1 of reference [12]).

Unfortunately, cause-specific results of the redistribution models are not available, thus, it remains unknown how many of the estimated ACM deaths have been redistributed from which garbage code. However, heart failure deaths have been cited as one out of three garbage codes which were redistributed to ACM in the GBD study [12]. Further, heart failure deaths account for a substantial share of CVD deaths in civil registry data and several studies have proposed methods to redistribute heart failure deaths [13,16,23,35]. In brief, heart failure describes an impaired functioning of the heart muscle and is an intermediate state between death and the actual underlying cause, which can be CVDs (e.g., cardiomyopathy, ischemic heart disease) but also other non-communicable diseases such as chronic respiratory diseases, diabetes, or cirrhosis [12,35]. 

Results from our sensitivity analyses suggest that heart failure deaths may not be redistributed to ACM in the majority of countries included in this study. This is in line with previous studies showing only marginal—if any—increases of mortality from all cardiomyopathies after redistributing heart failure garbage codes [16,23]. In GBD, senility and atherosclerosis have been referred to as other garbage codes, which were redistributed to CVD, including ACM [12]. More details on the misclassification of cause of deaths codes should be provided in the GBD study to improve clinical care and cause of death coding practice. For ACM, this is particularly important as five out of six deaths may not be recognized.

### 4.4. Clinical Relevance

There is a large gap between registered and estimated deaths due to ACM but also due to all other cardiomyopathies. Primarily, a large number of deaths assigned with garbage codes may be the result of inaccurate cause of death coding. However, this gap could also be an indicator for suboptimal clinical care (detection, treatment) during a lifetime. To diagnose ACM, clinicians need to identify a dilated heart muscle, rule out other potential causes, and conduct an extensive assessment of the patients’ alcohol use [3,22]. However, the substantial stigma around alcohol dependence [36] may deter clinicians from such conversations with their patients, as reported in primary care [37]. Clinicians may further be discouraged to have discussions around alcohol with their patients because of several uncertainties with this topic, e.g., the differential impact on diseases—especially for cardiovascular diseases, for which both beneficial and detrimental effects have been observed (for an overview of risk functions, see reference [27]), the fact that there is no recognized “safe” level of alcohol consumption (for a recent discussion, see references [38,39,40]), or the lack of an international consensus in defining risky drinking [41].

However, even if all ACM cases were accurately identified in clinical practice (i.e., no garbage codes), the contribution of alcohol may still be underestimated if based on death certificates only [42]. In summary, vital statistics may be the best available data source to estimate ACM mortality to date. Yet, prospective studies on the relationship between alcohol intake and incidence of cardiomyopathy are required to yield more accurate mortality estimates.

### 4.5. Strengths and Limitations

This study used mortality data from 77 countries, representing the majority of high-income countries with highly accurate data from civil registries [9]. However, countries without such data are not represented in this study, most notably African and Asian countries. Thus, the presented results may not apply to non-Caucasian populations from low- and middle-income countries. Further, we restricted the sensitivity analyses to heart failure, which was cited as major source for garbage code redistribution for ACM. As ACM-specific results from the algorithm that redistributes garbage codes in the GBD 2017 study are not available, the impact of redistributing other garbage codes to ACM mortality could not be determined in this study. As the alcohol exposure data used in the GBD study are not available to the authors, we used data from a recent modeling study based on WHO collection and estimation of *per capita* consumption per country, which is considered the most valid estimate of overall alcohol exposure [43], and was validated by country representatives [44]. This has limited the breakup of mortality data to age groups, for which alcohol exposure data is available. 

## 5. Conclusions

GBD mortality estimates of ACM are implausible for adults aged 65 years or older, as they are incongruent with civil registry and alcohol exposure data in this age group. In order to produce more consistent ACM mortality estimates, the redistribution algorithms in the GBD study should be aligned with alcohol exposure data.

## Figures and Tables

**Table 1:** Registered and estimated deaths by disease category and sex.

GBD Disease Definition	Absolute Number of Deaths			Age-Standardized Mortality Rate ^1^		
	Women	Men	Both sexes	Women	Men	Both sexes
**Cardiovascular diseases**						
Registered ^2^	1,396,965	1,469,004	2,865,969	89.1	148.3	116.1
Estimated ^2^	2,459,266	2,386,248	4,845,514	154.9	239.3	193.6
Ratio ^3^	1.8	1.6	1.7	1.7	1.6	1.7
**Cardiomyopathy and myocarditis**						
Registered **^2^**	8,240	11,938	20,178	0.6	1.4	1.0
Estimated ^2^	66,730	75,616	142,346	4.4	8.0	6.1
Ratio ^3^	8.1	6.3	7.1	6.8	5.9	6.2
**Alcoholic Cardiomyopathy**						
Registered ^2^	538	3,345	3,883	0.1	0.4	0.2
Estimated ^2^	3,589	18,894	22,483	0.3	2.1	1.2
Ratio ^3^	6.7	5.6	5.8	5.8	5.4	5.3
**Cardiovascular garbage codes**						
Registered ^2^	963,461	780,529	1,743,990	58.4	77.1	67.2
Estimated ^2^	/	/	/	/	/	/
Ratio ^3^	/	/	/	/	/	/
**Heart failure garbage codes**						
Registered ^2^	283,222	209,033	492,255	15.8	20.0	17.8
Estimated ^2^	/	/	/	/	/	/
Ratio ^3^	/	/	/	/	/	/

Note: Based on *n* = 77 countries, see Supplementary Table S1 for a detailed list of the included countries. ^1^ Age-standardized number of deaths per 100,000 adults. ^2^ All deaths obtained from in civil registries [18] or as estimated in the GBD 2017 study [11]. ^3^ Ratio of estimated to registered deaths.

**Table 2:** Correlation of registered and estimated ACM crude mortality rates (deaths per 100,000 people) for all adults and by sex and age groups.

	Women	Men
**All Adults**	0.796 (0.697 to 0.866) **	0.917 (0.872 to 0.946) **
**By age group**		
15–19	NA	NA
20–24	NA	0.968 (0.95 to 0.979) **
25–29	0.212 (−0.012 to 0.416)	0.985 (0.977 to 0.991) **
30–34	0.42 (0.217 to 0.589) **	0.988 (0.981 to 0.992) **
35–39	0.731 (0.607 to 0.821) **	0.956 (0.932 to 0.972) **
40–44	0.779 (0.672 to 0.854) **	0.963 (0.942 to 0.976) **
45–49	0.955 (0.93 to 0.971) **	0.941 (0.908 to 0.962) **
50–54	0.956 (0.932 to 0.972) **	0.764 (0.652 to 0.844) **
55–59	0.815 (0.723 to 0.879) **	0.777 (0.67 to 0.853) **
60–64	0.899 (0.845 to 0.935) **	0.901 (0.848 to 0.936) **
65–69	0.305 (0.087 to 0.495) *	0.545 (0.366 to 0.685) **
70–74	0.513 (0.326 to 0.661) **	0.73 (0.605 to 0.82) **
75–79	0.184 (−0.042 to 0.391)	0.618 (0.458 to 0.74) **
80–84	0.099 (−0.127 to 0.316)	0.158 (−0.069 to 0.369)
85–99	0.025 (−0.2 to 0.247)	0.136 (−0.091 to 0.349)

Note: Based on most recent available data from *n* = 77 countries, see Supplementary Table S1 for a detailed list of the included countries; * *p* < 0.01; ** *p* < 0.001; NA = correlations could not be calculated due to zero registered deaths.

**Figure A1:**
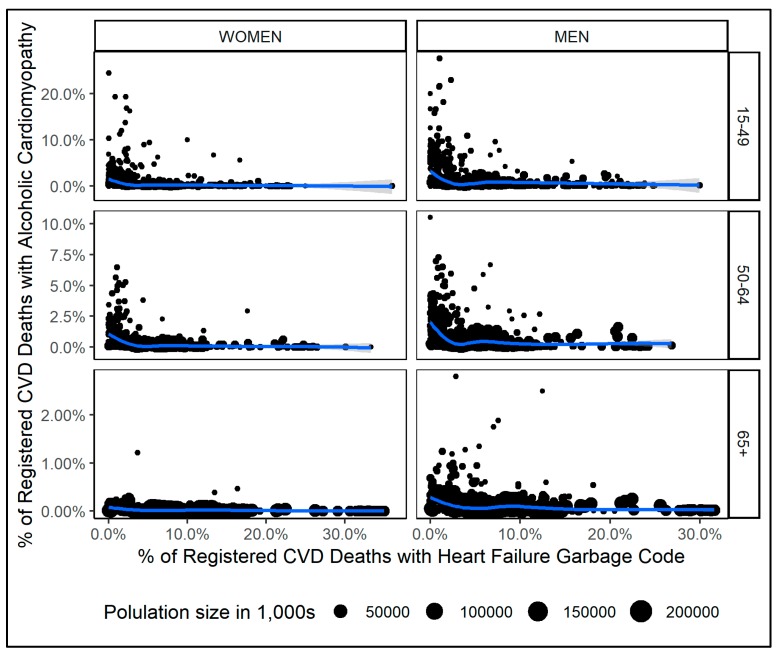
Scatter plots of % HF garbage code deaths and % ACM deaths among all CVD deaths by sex and age group; the blue line denotes a weighted smoothing function of the two variables.

## Supplementary Materials

The following are available online at https://www.mdpi.com/2077-0383/8/8/1137/s1, Supplementary Table S1: Country-level data for key variables including source years. The dataset generated and analyzed during this study is available in the figshare repository, https://figshare.com/articles/Mortality_from_alcoholic_cardiomyopathy/7624766 (DOI: 10.6084/m9.figshare.7624766).

## Appendix A

### Appendix A.1. Methods

#### Appendix A.1.1. Definition of Cause of Death Categories

In Appendix Table A1, the disease definitions for all cardiovascular diseases (including garbage codes), cardiovascular diseases (excluding garbage codes), all cardiomyopathies, alcoholic cardiomyopathy, and cardiovascular as well as heart failure garbage codes are presented. The table lists ICD-10 cause of deaths codes matched with mortality data obtained from the World Health Organization.

**Table A1:** Cause of death definition.

GBD Cause of Death Definition	ICD-10 Cause of Death Codes	Short Term Used in Manuscript
All cardiovascular diseases (sum of garbage and non-garbage codes)	***Non-garbage:*** B33.2, G45-G46.8, I01-I01.9, I02.0, I05-I09.9, I11-I11.9, I20-I25.9, I28-I28.8, I30-I31.1, I31.8-I37.8, I38-I41.9, I42.1-I42.8, I43-I43.9, I47-I48.9, I51.0-I51.4, I60-I63.9, I65-I66.9, I67.0-I67.3, I67.5-I67.6, I68.0-I68.2, I69.0-I69.3, I70.2-I70.8, I71-I73.9, I77-I83.9, I86-I89.0, I89.9, I98, K75.1 ***Garbage:*** *Level-1:* I26-I26.9, I31.2-I31.4, I37.9, I46-I46.9, I50-I50.9, I51.7, I67.4, I76, I95-I95.1, I95.8-I95.9, *Level-2:* I10-I10.9, I15-I15.9, I27-I27.0, I27.2-I27.9, I28.9, I70-I70.1, I70.9, I74-I75.8 *Level-3:* I00.0, I03-I04., I14-I14., I16-I19, I29-I29.9, I44-I45.9, I49-I49.9, I51, I51.6, I51.8-I59, I90-I94, I96-I96.9, I98.4-I98.8, I99 *Level-4:* I42-I42.0, I42.9, I51.5, I64-I64.9, I67, I67.8-I68, I68.8-I69, I69.4-I69.9	All CVD
Cardiovascular diseases	B33.2, G45-G46.8, I01-I01.9, I02.0, I05-I09.9, I11-I11.9, I20-I25.9, I28-I28.8, I30-I31.1, I31.8-I37.8, I38-I41.9, I42.1-I42.8, I43-I43.9, I47-I48.9, I51.0-I51.4, I60-I63.9, I65-I66.9, I67.0-I67.3, I67.5-I67.6, I68.0-I68.2, I69.0-I69.3, I70.2-I70.8, I71-I73.9, I77-I83.9, I86-I89.0, I89.9, I98, K75.1	CVD
Cardiomyopathy and myocarditis	B33.2, I40-I41.9, I42.1-I42.8, I43-I43.9, I51.4	All cardiomyopathies
Alcoholic cardiomyopathy	I42.6	ACM
Garbage codes in ICD-10 category of circulatory diseases	*Level-1:* I26-I26.9, I31.2-I31.4, I37.9, I46-I46.9, I50-I50.9, I51.7, I67.4, I76, I95-I95.1, I95.8-I95.9, *Level-2:* I10-I10.9, I15-I15.9, I27-I27.0, I27.2-I27.9, I28.9, I70-I70.1, I70.9, I74-I75.8 *Level-3:* I00.0, I03-I04., I14-I14., I16-I19, I29-I29.9, I44-I45.9, I49-I49.9, I51, I51.6, I51.8-I59, I90-I94, I96-I96.9, I98.4-I98.8, I99 *Level-4:* I42-I42.0, I42.9, I51.5, I64-I64.9, I67, I67.8-I68, I68.8-I69, I69.4-I69.9	CVD garbage codes
Heart failure garbage codes	I50	HF garbage code

Note: Mapping of ICD-10 codes to disease category obtained from GBD 2017 study.

#### Appendix A.1.2. Description of Sensitivity Analyses

In the GBD study, a redistribution model is applied to estimate so called redistribution proportions, which are employed for redistributing garbage coded deaths to other diseases. In this study, we performed a similar model building on the hypotheses that among all cardiovascular deaths, the proportion of deaths with heart failure (HF) garbage codes is negatively associated with the proportion of ACM deaths.

For the sensitivity analyses, we used all mortality data from all available years, i.e. from 823 country-years (for details, see Supplementary Table S1). The denominator for calculating HF garbage code and ACM proportions was ‘all CVD’ and was calculated from the sum of all registered deaths from CVD and CVD garbage codes (for definition, see Appendix Table A1). Prior to building regression models, the data structure of the dependent variable (% of ACM deaths) was examined using scatter plots and bivariate correlations with % of HF garbage code deaths and alcohol exposure (for results see Appendix Table A2).

**Table A2:** Bivariate correlations of % HF garbage codes and % ACM mortality.

		15–49 Year Olds			50–64 Year Olds			65+ Year Olds		
		% ACM	% HF Garbage Codes	APC	% ACM	% HF Garbage Codes	APC	% ACM	% HF Garbage Codes	APC
***Women***	% ACM	1			1			1		
	% HF Garbage codes	−0.15	1		−0.24	1		−0.19	1	
	APC	0.26	−0.17	1	0.34	−0.14	1	0.21	−0.01	1
***Men***	% ACM	1			1			1		
	% HF Garbage codes	−0.24	1		−0.29	1		−0.21	1	
	APC	0.30	−0.15	1	0.35	−0.15	1	0.21	−0.10	1

Note: ACM = Alcoholic cardiomyopathy. HF = Heart failure. APC = Alcohol per capita consumption. Bivariate correlations based on mortality data from 823 country-years with available civil registry mortality data. Proportion of deaths are calculated from the denominator of all CVD deaths (including garbage codes).

Between ACM and heart failure, the distribution of deaths across age groups were substantially different. Among 50 to 64 year olds, the largest share of ACM deaths (47%) but only a fraction of HF garbage code deaths (7%) have been registered. This pattern reversed for the older age groups (65 years and older), among which 29% of all ACM deaths but 91% of all deaths assigned with HF garbage codes have been registered. Further and as illustrated in Appendix Figure A1, the association of % ACM deaths and % HF garbage code deaths varied largely across sex and age. Consequently, all regression models were stratified by sex and age group (15–49, 50–64, 65+).

For each stratum, we fitted the following models: (1A) a fractional response model with a linear combination of both covariates (% HF garbage code deaths and alcohol per capita consumption) and allowing for random intercepts for each country; (1B) a Poisson regression where the dependent variable was multiplied with 10,000 and rounded to the nearest integer and with the same covariate structure as in (1A); (2A) a fractional response regression as in 1A but with an additional set of 3rd order orthogonal polynomials of % HF garbage code deaths (as suggested by scatter plots, see Appendix Figure A1); (2B) a Poisson regression as in 1B but with an additional set of 3rd order orthogonal polynomials of % HF garbage code deaths. Poisson regression models (1B and 2B) were found to be superior to fractional response models in terms of data fit as assessed using *R*² and plotting fitted and observed data. Subsequently, Poisson models with linear and polynomial covariates were compared within each stratum using analysis of deviance tests. For most strata, model 2B (random effects Poisson regression with 3rd order polynomials of % HF garbage code deaths) were found the best fitting model (Chi²-tests: *p* < 0.001 for all tests), except young females, for which model 1B indicated best fit (random effects Poisson regression with linear combination of covariates).

For the random effects Poisson regression models, the equation is written as generalized linear model and was performed by sex and three age groups (15–49, 50–64, 65+):

$$ACM_{c}=\;f\left(\alpha +\;\beta_{1}HF+\;\beta_{2}HF^{2}+\;\beta_{3}HF^{3}+\beta_{4}APC+\gamma_{c}+\varepsilon_{c}\right)$$

where:

f() = Poisson function with log link function and Poisson distributed data
ACM_c_ = percentage of all cardiovascular deaths which were coded to alcohol cardiomyopathy, by country
α = constant
HF = percentage of all cardiovascular deaths which were coded to heart failure
β_1_ = slope coefficient describing the association between HF and ACM_c_
β_2_ = slope coefficient describing the association between the polynomial HF^2^ and ACM_c_ (not included among young females)
β_3_ = slope coefficient describing the association between the polynomial HF^3^ and ACM_c_ (not included among young females)
APC = Alcohol per capita consumption
β_4_ = slope coefficient describing the association between APC and ACM_c_
γ_r_ = country-specific random intercept
ε_c_ = standard error

For comparison, the regression equation used in the GBD study to calculate redistribution proportions is as follows (for details, see Appendix 1 of [12]):

$$TG_{crt}=\;\alpha +\;\beta_{1}Gar_{crt}+\;\beta_{2}Age_{crt}Gar_{crt}+\;\theta_{r}Gar_{crt}+\gamma_{r}+\varepsilon_{ct}$$

where:

TG_crt_ = percentage of deaths within the given garbage code’s universe which were coded to a
given target group, by country
α = constant
Gar_crt_ = percentage of deaths within the given garbage code’s universe which were coded to a given set of garbage codes
β_1_ = slope coefficient describing the association between Gar_crt_ and TG_crt_
β_2_ = slope coefficient describing the association between the interaction Age_crt_ and Gar_crt_
γ_r_ = region-specific random intercept (or super-region if the random effect on region is not
significant)
θ_r_ = region-specific random slope (or super-region if the random effect on region is not
significant)
ε_ct_ = standard error, normally distributed and calculated by bootstrapping

There are two main differences between the GBD redistribution model and the model presented in this study. First, we accounted for alcohol exposure as the core determinant for ACM and second, allowed for a non-linear relationship between HF garbage code and ACM mortality proportions.

### Appendix A.2. Results

#### Appendix A.2.1. Mortality Rates of Registered and Estimated Deaths

In Appendix Table A3, Table A4 and Table A5, the mortality rates of cardiovascular diseases, all cardiomyopathies, and alcoholic cardiomyopathy are presented by sex and age. The table also includes the ratio of estimated to registered mortality rates, which are largely constant for cardiovascular diseases but increase with age for all cardiomyopathies and alcoholic cardiomyopathy.

**Table A3:** Mortality rates of registered and estimated deaths of *alcoholic cardiomyopathies* by sex and age.

	Women			Men			Both Sexes		
	Registered	Estimated	Ratio	Registered	Estimated	Ratio	Registered	Estimated	Ratio
15–19	0.00	0.01	NA	0.00	0.03	NA	0.00	0.02	NA
20–24	0.00	0.02	NA	0.01	0.06	6.6	0.00	0.04	8.5
25–29	0.01	0.03	3.9	0.04	0.16	3.5	0.03	0.10	3.6
30–34	0.01	0.06	5.3	0.11	0.36	3.3	0.06	0.21	3.5
35–39	0.01	0.09	6.7	0.23	0.68	3.0	0.12	0.39	3.2
40–44	0.05	0.17	3.4	0.34	1.25	3.7	0.19	0.71	3.7
45–49	0.10	0.31	3.1	0.56	2.04	3.6	0.33	1.17	3.6
50–54	0.10	0.46	4.7	0.83	3.10	3.7	0.46	1.76	3.8
55–59	0.16	0.73	4.6	1.00	4.70	4.7	0.57	2.66	4.7
60–64	0.22	0.95	4.2	1.16	6.02	5.2	0.67	3.38	5.0
65–69	0.14	1.03	7.2	1.14	6.88	6.0	0.61	3.79	6.2
70–74	0.14	1.15	8.3	0.95	7.96	8.4	0.51	4.26	8.4
75–79	0.09	1.32	15.5	0.69	8.80	12.7	0.35	4.58	13.1
80–84	0.09	1.44	15.4	0.56	8.59	15.2	0.28	4.32	15.2
85–99	0.05	2.39	45.5	0.32	9.50	29.4	0.14	4.72	33.4

Note: Mortality rates = deaths per 100,000 population. Ratio = ratio of estimated to registered deaths. Mortality data obtained from *n* = 77 countries (see Supplementary Table S1).

**Table A4:** Mortality rates of registered and estimated deaths of cardiovascular diseases by sex and age.

	Women			Men			Both Sexes		
	Registered	Estimated	Ratio	Registered	Estimated	Ratio	Registered	Estimated	Ratio
15–19	1.3	2.8	2.2	2.1	4.1	1.9	1.7	3.5	2.0
20–24	1.9	4.1	2.1	3.8	6.6	1.7	2.9	5.3	1.8
25–29	3.0	5.8	1.9	6.1	10.3	1.7	4.6	8.1	1.8
30–34	4.9	9.2	1.9	10.6	17.5	1.7	7.7	13.4	1.7
35–39	8.7	15.3	1.8	19.0	30.0	1.6	13.8	22.7	1.6
40–44	15.5	26.0	1.7	36.0	53.9	1.5	25.7	39.9	1.6
45–49	26.7	43.9	1.6	63.6	94.3	1.5	45.0	68.9	1.5
50–54	43.4	71.2	1.6	109.8	162.1	1.5	76.0	116.0	1.5
55–59	70.1	114.5	1.6	176.3	265.9	1.5	121.8	188.2	1.6
60–64	112.6	188.1	1.7	270.1	414.3	1.5	188.0	296.4	1.6
65–69	181.5	304.2	1.7	386.3	600.0	1.6	278.1	443.8	1.6
70–74	320.1	549.8	1.7	588.2	950.7	1.6	442.5	732.9	1.7
75–79	604.2	1039.4	1.7	954.0	1552.5	1.6	756.8	1263.3	1.7
80–84	1162.5	2008.5	1.7	1632.0	2699.4	1.6	1351.4	2286.5	1.7
85–99	3112.1	5667.5	1.8	3538.3	6178.5	1.8	3251.8	5835.0	1.8

Note: Mortality rates = deaths per 100,000 population. Ratio = ratio of estimated to registered deaths. Mortality data obtained from *n* = 77 countries (see Supplementary Table S1).

**Table A5:** Mortality rates of registered and estimated deaths of *all cardiomyopathies* by sex and age.

	Women			Men			Both Sexes		
	Registered	Estimated	Ratio	Registered	Estimated	Ratio	Registered	Estimated	Ratio
15–19	0.1	0.3	2.3	0.3	0.6	2.5	0.2	0.5	2.4
20–24	0.1	0.4	4.1	0.2	0.8	3.5	0.2	0.6	3.7
25–29	0.2	0.5	3.3	0.4	1.2	3.1	0.3	0.8	3.2
30–34	0.2	0.6	3.7	0.5	1.6	3.4	0.3	1.1	3.5
35–39	0.3	0.9	3.6	0.8	2.4	3.1	0.5	1.6	3.2
40–44	0.3	1.2	3.7	1.0	3.6	3.7	0.7	2.4	3.7
45–49	0.5	1.7	3.7	1.4	5.2	3.7	1.0	3.5	3.7
50–54	0.6	2.5	4.2	1.9	7.6	4.0	1.3	5.0	4.0
55–59	0.8	3.9	4.8	2.4	11.1	4.7	1.6	7.4	4.7
60–64	1.1	5.5	5.1	2.8	14.7	5.3	1.9	9.9	5.2
65–69	1.3	8.1	6.4	3.2	19.1	6.0	2.2	13.3	6.1
70–74	1.9	13.4	7.0	3.4	26.9	8.0	2.6	19.6	7.6
75–79	2.9	25.2	8.7	4.5	42.1	9.4	3.6	32.6	9.1
80–84	5.5	50.4	9.2	6.1	67.4	11.1	5.7	57.3	10.0
85–99	13.2	150.9	11.5	13.4	151.1	11.3	13.2	151.0	11.4

Note: Mortality rates = deaths per 100,000 population. Ratio = ratio of estimated to registered deaths. Mortality data obtained from *n* = 77 countries (see Supplementary Table S1).

#### Appendix A.2.2. Sensitivity Analyses: ACM and Heart Failure Deaths

Model results of random effects Poisson regressions are presented in Appendix Table A4. Data fit of regression models was measured in the correlation between observed and fitted data, which was satisfactory (greater than 0.7) in the young- and middle-age strata and poorer in the oldest age group (correlation below 0.5). Further, the variation in the dependent variable (% of ACM deaths) was lowest in the oldest age group, most pronounced among females.

In post-hoc analyses, we examined characteristics of countries where the proportion of CVD deaths assigned with HF garbage codes was lower than 5%. A low share of HF garbage code deaths has been identified in 226 out of 823 country-years (27%) and has been associated with higher age-standardized estimated and registered mortality rates for CVD, all cardiomyopathies and ACM, as well as a lower population size (*p* < 0.001 for females and males, obtained from Poisson regressions). Further, a low share of HF garbage code deaths was linked to higher alcohol exposure among males (*p* < 0.001 from linear regression) but not among females (*p* = 0.019 from linear regression).

**Table A6:** Results of regression models on % ACM deaths among all CVD deaths by age and sex.

	15- to 49-Years-Old		50- to 64-Years-Old		65 Years or Older	
	Women	Men	Women	Men	Women	Men
**Fixed effects (standard error)**						
Intercept	1.11 (0.38) *	3.43 (0.22) **	-0.03 (0.54)	3.03 (0.2) **	−1.81 (0.48) **	1.24 (0.26) **
First order polynomial of % HF garbage code deaths	7.87 (0.48) **	8.09 (0.27) **	8.19 (0.53) **	9.05 (0.21) **	6.94 (1.08) **	9.39 (0.31) **
Second order polynomial of % HF garbage code deaths ^1^	/	1.96 (0.2) **	4.01 (0.41) **	2.06 (0.15) **	2.75 (1.26)	2.49 (0.27) **
Third order polynomial of % HF garbage code deaths ^1^	/	−3.3 (0.21) **	−3.03 (0.4) **	−3.92 (0.13) **	−4.63 (0.71) **	−2.21 (0.19) **
Alcohol *per capita* consumption	0.02 (0.01)	0.02 (0.002) **	−0.02 (0.01)	0.01 (0.002) **	−0.04 (0.02)	−0.01 (0.003) **
**Random effects**						
Standard deviation of country-level intercepts	3.07	1.88	3.73	1.72	3.29	2.23
*R*-square ^2^	0.772	0.815	0.868	0.732	0.333	0.475
Mean (standard deviation) of dependent variable	0.7% (2.1%)	1.5% (2.9%)	0.3% (0.7%)	0.8% (1.2%)	0.02% (0.06%)	0.1% (0.2%)

Note: All results from Poisson regressions (outcome multiplied by 10,000 and rounded to nearest integer) with random intercepts for each country. All models based on *n* = 77 countries (823 country-years) with available mortality data from civil registries. ACM = alcoholic cardiomyopathy. CVD = Cardiovascular disease. HF = Heart failure. ^1^ The variable was centered around the sex- and age-specific mean. ^2^ *R*-square = square of the correlation coefficient between the actual and fitted values of the dependent variable. * *p* ≤ 0.01; ** *p* ≤ 0.001
